# A Comparative Study on the Mechanism of Delayed-Type Hypersensitivity Mediated by the Recombinant *Mycobacterium tuberculosis* Fusion Protein ESAT6-CFP10 and Purified Protein Derivative

**DOI:** 10.3390/ijms242316612

**Published:** 2023-11-22

**Authors:** Xiaonan Guo, Weixin Du, Junli Li, Jiaxin Dong, Xiaobing Shen, Cheng Su, Aihua Zhao, Yongge Wu, Miao Xu

**Affiliations:** 1 National Engineering Laboratory for AIDS Vaccine, School of Life Sciences, Jilin University, Changchun 130012, China; xnguojlu@163.com; 2 Division of Tuberculosis Vaccine and Allergen Products, Institute of Biological Product Control, National Institutes for Food and Drug Control, Beijing 102629, China; duwxin@126.com (W.D.); nifdclijunli@163.com (J.L.); dongjx000@163.com (J.D.); shengt99@163.com (X.S.); jhzz1225@hotmail.com (C.S.); tbtestlab@nifdc.org.cn (A.Z.)

**Keywords:** tuberculosis, EC, PPD, erythema, induration, DTH

## Abstract

While purified protein derivative (PPD) is commonly used as skin diagnostic reagent for tuberculosis (TB) infection, it cannot distinguish effectively Bacillus Calmette–Guérin (BCG) vaccination from *Mycobacterium tuberculosis* (MTB) complex and nontuberculous mycobacteria infection. The new skin reagent ESAT6-CFP10 (EC) has favorable sensitivity and specificity, which can overcome limitations associated with PPD. At present, EC skin test reactions are mainly characterized by erythema, while PPD mainly causes induration. We conducted a comparative study on the potential differences between EC-induced erythema and PPD-induced induration using a *guinea pig* model. The size of EC-dependent erythema was similar to that of PPD-induced induration, and an inflammatory response characterized by the infiltration of monocytes, macrophages and lymphocytes, as well as tissue damage, appeared at the injection site. The lymphocytes included CD4^+^ T and CD8^+^ T cells, which released *IFN-γ* as the main cytokine. Both EC erythema and PPD induration could lead to increased levels of acute-phase proteins, and the differential pathways were similar, thus indicating that the main induced immune pathways were similar. The above results indicated that erythema produced by EC could generate the main delayed-type hypersensitivity (DTH) response characteristic of PPD induration, thereby suggesting that erythema might also have a certain diagnostic significance and provide a possible theoretical basis for its use as a diagnostic indicator for detecting MTB infection.

## 1. Introduction

Tuberculosis (TB) is caused by *Mycobacterium tuberculosis* (MTB) and has high mortality rates [1,2]. The World Health Organization (WHO) reported 10.6 million new cases in 2021, resulting in a total of 1.6 million deaths [3]. Further, the population with latent tuberculosis infection (LTBI), a potential database of TB patients, is nearly 2 billion, and approximately 5% to 10% of it will develop active TB, with most people diagnosed within two years after infection [4,5,6]. As China has the highest prevalence of LTBI, affecting approximately 350 million people, the TB epidemic in China is difficult to control [7]. Therefore, an effective diagnosis of LTBI could serve as a main strategy for the prevention, control and elimination of TB [8].

The tuberculin skin test (TST) is widely used as an important diagnostic tool for MTB infection, and the common reactive agent involved is purified protein derivative (PPD) [9]. PPD is a protein mixture that is purified from the filtrate of an MTB culture by precipitation with trichloroacetic acid and ammonium sulfate. The preparation is primarily composed of MTB proteins as well as different peptides and contains a small amount of polysaccharides, nucleic acids and lipids [10]. The TST is performed by injecting PPD into an individual’s forearm and observing the skin induration reaction after 48 h or 72 h from the injection. Although the TST has been the standard for the identification of MTB infection for the past century, it cannot effectively distinguish between MTB infection and either nontuberculous mycobacteria (NTM) infection or Bacillus Calmette–Guérin (BCG) vaccination due to the presence of homologous antigens in PPD [11,12]. Therefore, the TST suffers from high false positive rate and low specificity [13], and these limitations have led to the development of more effective immunodiagnostic tools to identify LTBI.

The recombinant tuberculosis fusion protein ESAT6-CFP10 (EC) is recommended by the WHO as a novel skin diagnostic reagent for the detection of MTB infection [14]. EC is a fusion protein which was constructed through genetic engineering and comprises MTB early secretory antigen target 6 (ESAT-6) and culture filtrate protein 10 (CFP-10) [15]. These genes are located in the region of difference (RD) 1 of MTB, which is absent in BCG as well as in most NTM [16] and thus can differentiate effectively between BCG vaccination, NTM infection and MTB infection [17,18]. In addition, EC has a good safety profile [15,19,20], high sensitivity and specificity [21,22,23] and a low detection cost and is easy to administer as well as to promote and use at the grassroots level; thus, it is of great significance for TB control [24]. At present, the clinical application of EC causes mainly erythema, whereas the traditional PPD skin test reaction is mainly induration [25,26]. The latter is primarily due to delayed-type hypersensitivity (DTH) to tuberculin PPD, mainly induced by T lymphocyte-mediated skin inflammation [27]. DTH presents two distinct stages. The first one is the sensitization stage, in which DTH-inducing antigens such as MTB are captured as well as processed by antigen-presenting cells (APCs) and expressed on the surface of APCs in the form of MHC-II/I peptide complexes, which are then presented to specific T cells for recognition and subsequent activation. The activated cells can actively proliferate and differentiate into sensitized T cells targeting the MTB antigen. The second one is the effect stage, when the body is exposed to the same antigen again, which induces the sensitized T cells to secrete a series of cytokines and chemokines, causing mononuclear/macrophage, neutrophil, and lymphocyte aggregation, local exudation, edema and other inflammatory damage [28]. Clinical studies showed that erythema, as a diagnostic indicator of the EC skin test, can improve the sensitivity of the test without affecting its specificity [25]. To provide a further theoretical basis for the use of clinical erythema as a diagnostic indicator for detecting MTB infection, we examined the potential differences related to the mechanism of DTH mediated by the erythema response of EC and the induration response of PPD in a *guinea pig* model sensitized with MTB.

## 2. Results

### 2.1. Skin Test Reaction

**Figure 1:**
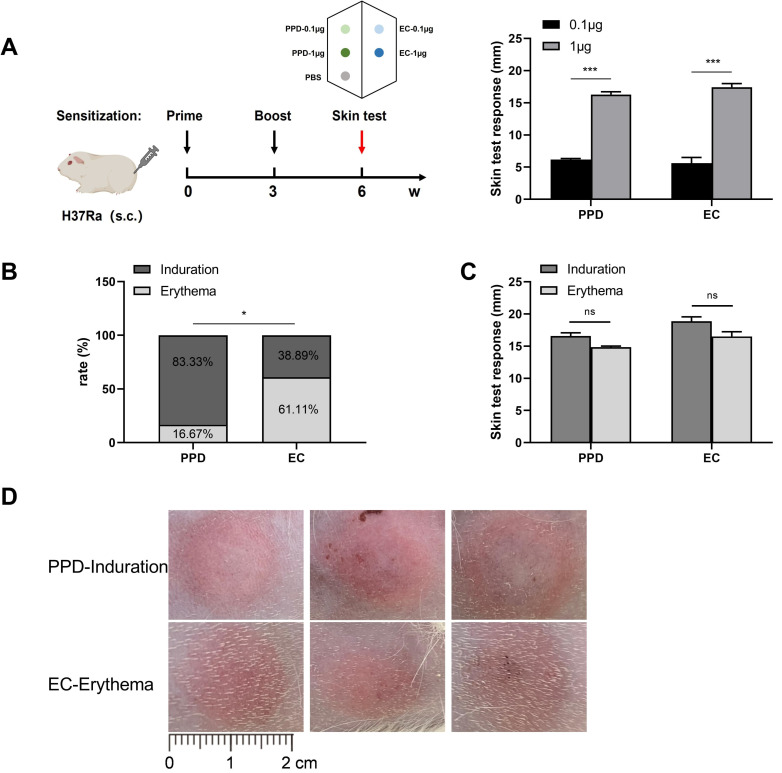
*Guinea pig* skin test scheme and skin test reaction. (**A**) Sensitization of *guinea pigs* and response sizes of the PPD and EC skin tests at different doses. H37Ra was used as the sensitizing strain and was sensitized twice with an interval of 3 weeks between the two injections. The skin tests were performed 3 weeks after the final sensitization. PPD/EC was injected intradermally (0.1 μg or 1 μg), the injection volume was 0.1 mL, and PBS was used as a negative control. (**B**) Incidence of PPD/EC induration and erythema at the 1 μg dose. (**C**) PPD/EC induration and erythema skin test responses at the 1 μg dose. (**D**) The size of the PPD induration and EC erythema responses at the dose of 1 μg. Data are expressed as mean ± SEM. * *p* < 0.05, *** *p* < 0.001 were considered to indicate significant differences; ns indicates no significant difference (*n* = 18).

First, *guinea pigs* (*n* = 18) were injected with 0.1 μg or 1 μg of the PPD/EC reagent, and the size of the skin test reaction was observed 24 h after the injection. The skin test responses of PPD and EC were found to be weak at the 0.1 μg dose, and there was no significant difference between PPD (6.2 ± 0.18 mm) and EC (5.1 ± 0.77 mm) (Figure 1A). Interestingly, obvious induration or erythema appeared at the PPD/EC dose of 1 μg, and there was no significant difference between PPD (16.3 ± 0.45 mm) and EC (17.4 ± 0.58 mm), but both were significantly larger than after the injection of 0.1 μg of reagent. The PBS injection site did not show any kind of skin reaction in all *guinea pigs*. The results indicated that PPD induration appeared in 15/18 animals (incidence of 83.33%), while EC induration was observed in 7/18 animals (incidence of 38.89%). The Fisher test revealed that the difference in the incidence of PPD and EC induration was statistically significant (*p* = 0.0153) (Figure 1B). PPD mainly caused induration, while EC mainly induced erythema. The induration reaction (PPD, 16.6 ± 0.51 mm; EC, 18.9 ± 0.70 mm) in response to the same skin test reagent was discovered to be slightly higher than the erythema reaction (PPD: 14.8 ± 0.17 mm, EC: 16.5 ± 0.73 mm), but there was no significant difference (Figure 1C). A separate comparison between PPD induration and EC erythema demonstrated that the two skin test reactions were similar in size (Figure 1D).

### 2.2. Pathological Changes in the Skin Test Site in Infected Guinea Pigs

**Figure 2:**
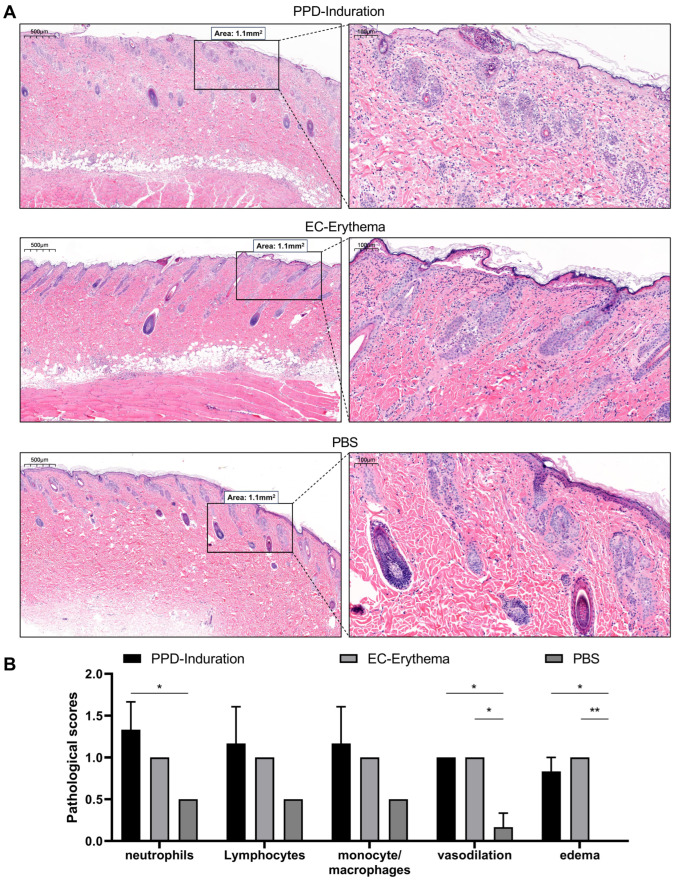
The various pathological changes and scores at the skin injection site of *guinea pigs* infected with MTB H37Ra. (**A**) Pathological changes induced by PPD induration, EC erythema and PBS after intradermal injection in *guinea pigs* (scale bars = 500 µm/100 µm). (**B**) Pathological scores of PPD induration, EC erythema and PBS. The black box indicates that the arbitrary interception area under a low-power mirror is 1.1 mm^2^. The data are shown as mean ± SEM. Experimental groups vs. PBS control; * *p* < 0.05 or ** *p* < 0.01 (*n* = 3).

To investigate whether PPD induration and EC erythema induced the infiltration of identical types of immune cells and the activation of the inflammatory response, H&E staining was performed on the skin of *guinea pigs* at the injection site. The results revealed that the pathological manifestations of PPD induration and EC erythema were identical, mainly concentrated in the dermis, with local infiltration of neutrophils, lymphocytes and mononuclear phagocytes, sparse dermal collagen fiber bundles, demonstrated as edema, mild vasodilation, hyperkeratosis and keratosis in some regions of the epidermis. However, only occasional inflammatory cell infiltration was observed at the PBS injection site, and no significant reaction was noticed (Figure 2A). The semi-quantitative score of the above-mentioned dermal pathological changes indicated that cell infiltration induced by PPD induration was slightly higher than that caused by EC erythema, but the difference was not significant. Cell infiltration was significantly higher in both groups than in the PBS group; vasodilation as well as edema were also significantly higher than in the PBS group (Figure 2B).

### 2.3. Analysis of Inflammatory Cell Infiltration

**Figure 3:**
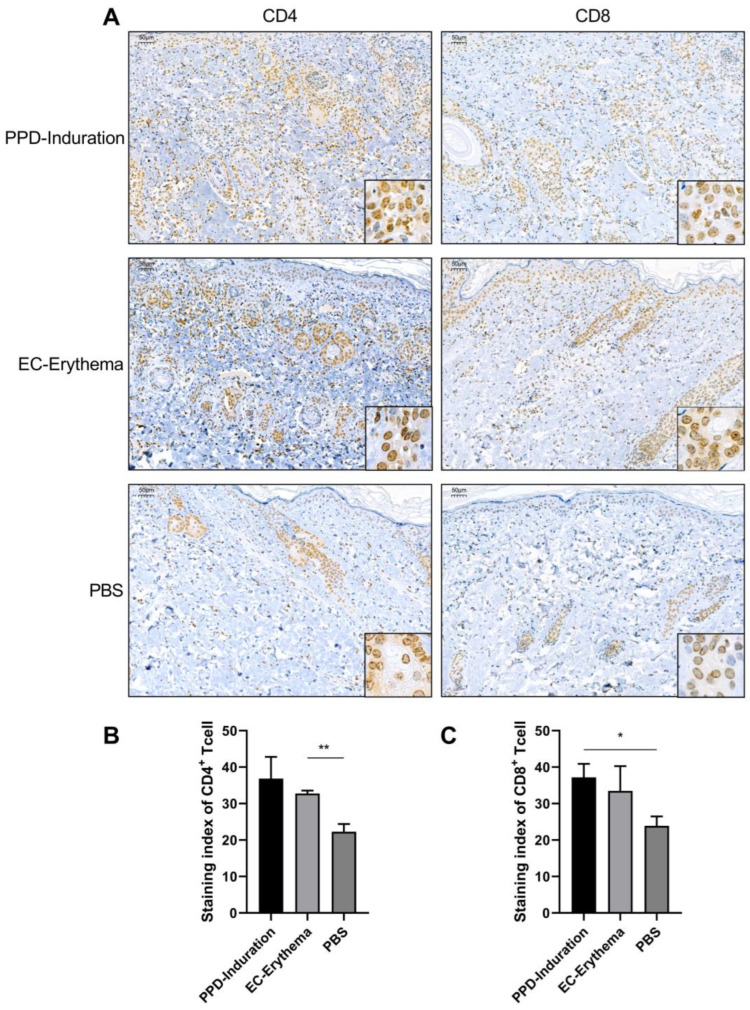
Immunohistochemical analysis of inflammatory cell infiltration at the skin injection sites in *guinea pigs* (scale bars = 50 µm). (**A**) Immunohistochemical analysis of PPD induration, EC erythema and control (PBS) in *guinea pigs*. *Guinea pig* skin tissues were stained with anti-CD4^+^ or anti-CD8^+^ monoclonal antibodies. Brown cells are positively stained cells. Staining index of CD4^+^ T (**B**) and CD8^+^ T (**C**) infiltrating cell. The data are shown as mean ± SEM. Experimental groups vs. PBS control; * *p* < 0.05, ** *p* < 0.01 (*n* = 3).

To determine the types of T lymphocytes that were found at the PPD induration and EC erythema response sites, immunohistochemical staining for CD4 and CD8 cells was performed at the injection sites. As shown in Figure 3A–C, T lymphocyte infiltration at the sites of PPD induration and EC erythema, including CD4^+^ T lymphocytes and CD8^+^ T lymphocytes, was noted to be higher in comparison to that in the PBS group. In particular, CD4^+^ T lymphocyte infiltration in the EC erythema group and CD8^+^ T lymphocyte infiltration in the PPD induration group were significantly different from those in the PBS group. In addition, T lymphocyte infiltration in PPD induration was slightly higher than in EC erythema, but there was no significant difference.

### 2.4. Analysis of Cytokine mRNA Expression Difference

**Figure 4:**
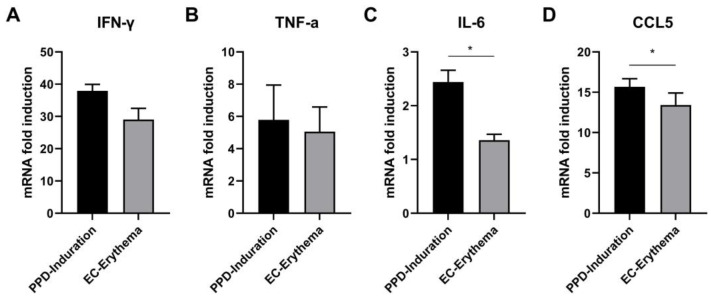
Cytokine mRNA expression in *guinea pig* skin test sites. The levels of *IFN-γ* (**A**), *TNF-α* (**B**), *IL-6* (**C**) and *CCL5* (**D**) mRNA in the PPD induration and EC erythema groups were compared with those in the PBS group. The values are expressed as mean ± SEM; PPD induration group vs. EC erythema group; * *p* < 0.05 (*n* = 4).

To further investigate the potential differences in cytokine secretion patterns, we analyzed variations in cytokine mRNA expression by RT-PCR. As shown in Figure 4, PPD induration and EC erythema revealed upregulated expression of *IFN-γ* and *TNF-α* compared with the PBS group (Figure 4A,B), while no increases were seen in *IL-4* expression, thus indicating that Th1 cytokines were mainly expressed in PPD induration and EC erythema. The *IFN-γ* level in PPD induration was found to be slightly higher than in EC erythema, but the differences in the levels of *IFN-γ* and *TNF-α* were not significant. At the same time, the inflammatory factors *IL-6* and chemokine *CCL5* were both detected, and it was noted that they were upregulated in both PPD induration and EC erythema compared to the control and that their levels were significantly higher in the PPD induration group than in the EC erythema group (Figure 4C,D).

### 2.5. Identification of Differentially Expressed Proteins (DEPs)

We next performed a TMT labeling-based proteomic analysis of the ventral skin samples from *guinea pigs* in the PPD induration, EC erythema and PBS control groups. Overall, 2168 different proteins were found. The length of the corresponding peptide segments of most of these proteins identified by mass spectrometry ranged from six to twenty-five distinct amino acid residues, thus indicating that the sample preparation was up to the standard and had been fully digested by trypsin (Figure S1A). Interestingly, it was observed that most proteins yielded more than one unique peptide, thereby indicating that these proteins were identified with high confidence (Figure S1B). The quality control results revealed that the protein identification in this study was accurate and provided a sound basis for the selection of DEPs.

**Figure 5:**
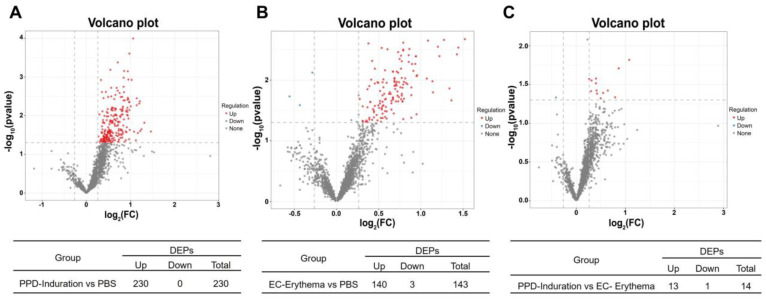
Identification of DEPs. Volcano plots of DEPs in PPD induration vs. PBS (**A**), EC erythema vs. PBS (**B**) and PPD induration vs. EC erythema (**C**). The *X*-axis represents the fold change in DEPs (log2 value), the *Y*-axis indicates the *p* value (−log10 value), the gray color represents non-significant differences, the red color indicates the upregulated proteins, and the blue color shows the down-regulated proteins.

In order to screen the DEPs between the different groups, protein fold change (FC) > 1.2 and *p* < 0.05 were used as potential discrimination criteria. As depicted in Figure 5A–C and Supplementary Tables S1–S3, 230 proteins were upregulated in the PPD induration group in comparison with the control PBS group. In the EC erythema group, 140 proteins showed upregulation, and 3 showed downregulation. In addition, compared with the EC erythema group, 13 proteins were upregulated, and 1 was down-regulated in the PPD induration group. On the whole, the PPD induration and EC erythema groups were found to be significantly different from the PBS group; DEPs in the PPD induration and EC erythema groups were less.

### 2.6. Gene Ontology (GO) Enrichment Analysis of DEPs

**Figure 6:**
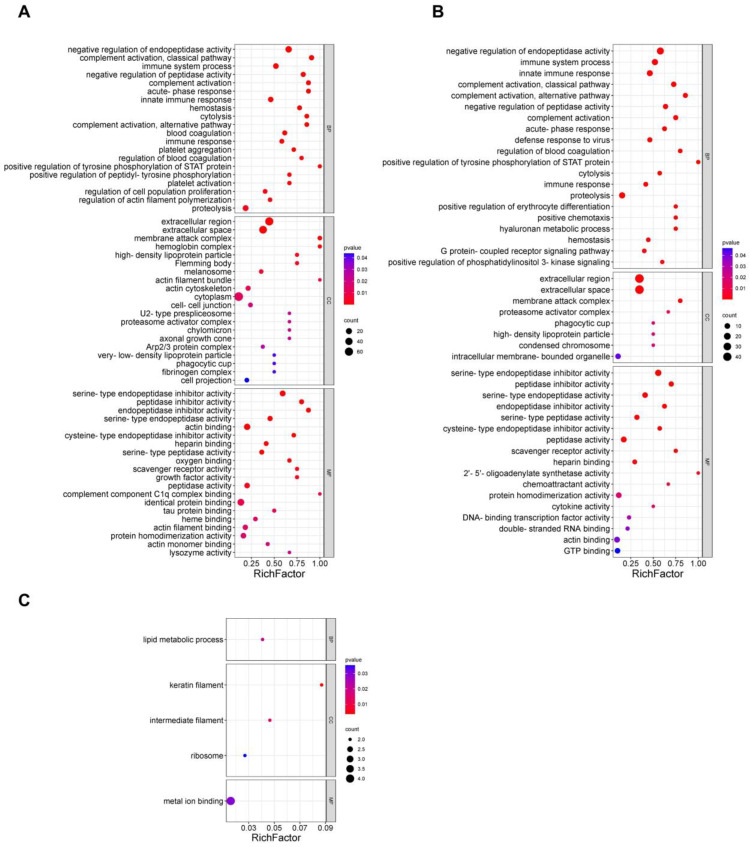
GO functional enrichment analysis of DEPs. Bubble plots of GO enrichment analysis in PPD induration vs. PBS (**A**), EC erythema vs. PBS (**B**) and PPD induration vs. EC erythema (**C**). The *X*-axis represents the enrichment factor value, and the *Y*-axis the GO terms. Colors indicate the *p*-values, with a darker color indicating more significant enrichment. Dot sizes indicate the number of DEPs. The first 20 results of the *p*-value were selected by default. If the number was less than 20, all the results were displayed.

Functional annotations of DEPs indicating the biological process (BP), cellular component (CC) and molecular function (MF) GO categories were investigated. As depicted in Figure 6A–C, the PPD induration/EC erythema groups were compared with the PBS group as regards biological processes, with DEPs mainly associated with functions such as negative regulation of endopeptidase activity, complement activation, acute phase reaction, hemostasis, participation in immune system reaction, positive regulation of tyrosine phosphorylation of signal transducer and activator of transcription (STAT) protein, and inflammatory response. Interestingly, it was noted that compared with the EC erythema group, the PPD induration group showed enrichment only in DEPs involved in the biological function of lipid metabolism. The PPD induration/EC erythema groups were compared with the PBS control as regards cellular components, including DEPs in extracellular regions and extracellular space, membrane attack complexes, etc. The PPD induration group was enriched only in keratin filaments, intermediate filaments and ribosomes in comparison to the EC erythema group. The PPD induration/EC erythema groups were compared with the PBS group as regards molecular function, including DEPs possessing diverse molecular functions such as serine-type endopeptidase inhibitor activity, serine-type endopeptidase activity and cytokine activity. However, only DEPs with the function of metal ion binding were enriched in the PPD induration group in comparison to the EC erythema group.

### 2.7. Kyoto Encyclopedia of Genes and Genomes (KEGG) Enrichment Analysis of DEPs

**Figure 7:**
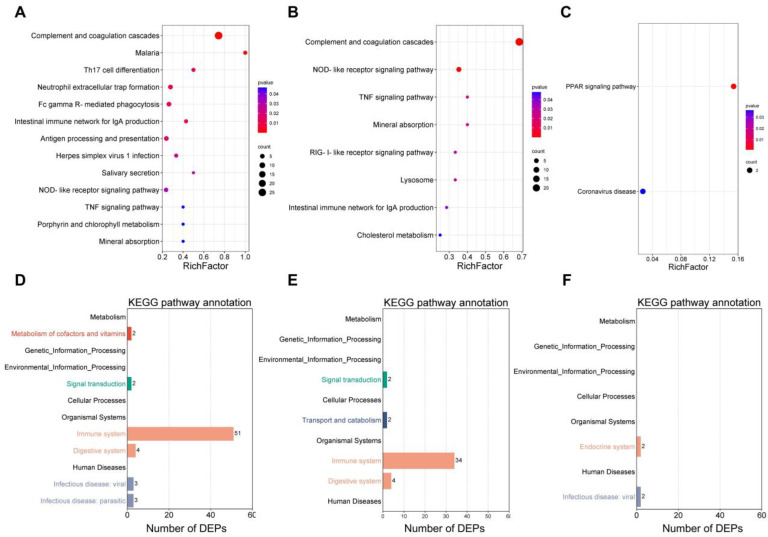
KEGG pathway enrichment analysis of the DEPs. Bubble plots of KEGG pathway enrichment analysis for PPD induration vs. PBS (**A**), EC erythema vs. PBS (**B**), PPD induration vs. EC erythema (**C**). The *X*-axis indicates the degree of enrichment, and the *Y*-axis the KEGG pathway. The *p*-values are indicated by color, with more intense colors representing greater enrichment. Dot sizes indicate the number of DEPs. Secondary classification of KEGG enrichment for PPD induration vs. PBS (**D**), EC erythema vs. PBS (**E**), PPD induration vs. EC erythema (**F**). The *X*-axis indicates the number of DEPs, and the *Y*-axis the KEGG pathways.

KEGG is an information network connecting various known molecular interactions, which is divided into seven main, distinct categories: metabolism, genetic information processing, environmental information processing, cellular processes, organic systems, human diseases, and drug development. KEGG enrichment analysis was used to examine the main biochemical, metabolic, as well as signal transduction pathways involved in PPD induration and EC erythema compared to the PBS control. It was noted that PPD induration/EC erythema compared with the control were enriched in DEPs involved mostly in the coagulation and complement cascades, NOD-like receptor signaling and TNF signaling (Figure 7A,B). Interestingly, in comparison with the EC erythema group, the DEPs in the PPD induration group were mainly involved in the PPAR signaling pathway, which is chiefly involved in lipid metabolism (Figure 7C). The enrichment pathways were classified, and the results are depicted in Figure 7D–F. It was observed that, compared with PBS, PPD induration/EC erythema DEPs were mainly involved in immune response, signal transduction and digestive system function. However, compared with the EC erythema group, DEPs in the PPD induration group were mainly related to the endocrine system, not to immune pathways.

## 3. Discussion

Recombinant MTB fusion protein EC, the creation tuberculin skin test (C-TST), has been approved for use by WHO. At present, EC mainly causes erythema in the skin test in clinical settings [25]. This study explored the differences in the mechanism of EC-induced erythema and PPD-induced induration based on five distinct aspects: skin test size, pathological changes, infiltrating cell types, differences in cell mRNA expression and DEPs.

We established a preclinical model in *guinea pigs* sensitized with the H37Ra strain. It was observed that the PPD induration and EC erythema response sizes and pathological changes were similar at the 1 µg dose of the reagent. Immunohistochemical analysis of the types of infiltrated T lymphocytes revealed that CD4^+^ and CD8^+^ T cells were primarily involved in the reaction, and their number was similar. CD4^+^ T cells serve as key factors in the DTH response. Protein antigens are taken up by APCs such as macrophages, Langerhans cells and dendritic cells through various exogenous pathways and after processing, antigen polypeptides are presented to sensitized CD4^+^ T cells in the form of MHC-Class II molecules. Thereafter, activated CD4^+^ T cells can release different cytokines, mainly *IFN-γ* and *TNF-α*. *IFN-γ*, an important amplification factor of the DTH response, can act on APCs to stimulate the expression of MHC-II molecules and thereby improve the efficiency of antigen presentation to local CD4^+^ T cells. It can also activate macrophages or CD8^+^ T cells, which can then directly damage tissues. *TNF-α* was reported to enhance the expression of endothelial cell adhesion molecules, resulting in neutrophil and monocyte aggregation, thus expanding the inflammatory response. At present, accumulating evidence indicates that antigens are processed and presented to CD8^+^ T cells during DTH [29,30], and exogenous antigens can be presented by targeting the endogenous pathways [31]. Our data also showed robust activation of CD4^+^ T and CD8^+^ T in PPD induration- and EC erythema-induced DTH reactions, indicating that specific antigenic peptides can be processed and presented on MHC-I and MHC-II molecules after antigen treatment by APCs.

The difference in cytokine induction in EC erythema and PPD induration was determined by analyzing the expression of the mRNA of various cytokines at the DTH response site in *guinea pigs*. The data revealed that both reactions could induce the same type of immune response, which could lead to the upregulation of both *IFN-γ* and *TNF-α* expression, while the *IL-4* level did not increase. The immune response could be a Th1-biased cellular response, and these findings are identical to those of a study by Yang et al. [30], who also observed increases in Th1 cytokines such as *IFN-γ* and *TNF-α,* with no evidence of Th2 cytokines such as *IL-10* at the different skin test sites. In addition, PPD induration was found to significantly increase the levels of the inflammatory factor *IL-6* and the chemokine *CCL5* compared to EC erythema. *IL-6* can activate the STAT3 protein and interact with the transcription factor NF-κB in the nucleus, which promotes inflammation and the transcription of acute-phase proteins, such as serum amyloid protein (SAA) as well as fibrinogen (FGA, FGB). *IL-6* can stimulate the activation of vascular endothelial growth factor (VEGF) or upregulate C5a receptors on endothelial cells, increasing both tissue damage and vascular permeability. *IL-6* can also promote tissue factor expression and trigger a clotting cascade, resulting in thrombin activation and fibrin clot formation. Fibrin is a highly insoluble protein polymer, and prior studies showed that the formation of fibrin could be related to the formation of induration [32]. *CCL5* (RANTES), a chemokine, can actively participate in the inflammatory response and specifically engage in chemokine T cell infiltration. The expression of *CCL5* in PPD induration was reported to be significantly higher than in EC erythema, which could increase CD4^+^ and CD8^+^ T infiltration, consistently with our immunohistochemical results.

In order to further understand the potential differences between the immune mechanisms of PPD induration and EC erythema, we conducted a proteomic detection of the DEPs and their enriched immune pathways. For this, TMT proteomics technology was employed, which uses 2, 6, 10 or 16 isotope labels to specifically label the amino group of peptides, and then tandem mass spectrometry was performed to compare the relative protein contents of the examined samples at the same time. The results revealed that in comparison with the PBS group, the PPD induration and EC erythema groups contained more DEPs, among which various acute-phase proteins, including binding globin (Hp), ceruloplasmin (CER), SAA, complement C3, C5, etc., presented significantly increased levels, indicating that PPD and EC injection formed an active inflammatory environment in the skin. In addition, the PPD induration group was also characterized by significant additional levels of FGA, FGB and fibronectin (FN) in comparison with the PBS group, which form fibrin, a water-insoluble protein that could be involved in the formation of induration. Interestingly, compared with the EC erythema group, the PPD induration group presented only 13 proteins that were significantly upregulated, including fatty acid binding protein 5 (FABP5), which, as a lipid transporter, can effectively bind fatty acids with high affinity, thus affecting lipid metabolism [33]. In addition, prior studies reported that the expression of FABP5 in the plasma of patients with femoral atherosclerosis was increased, and the expression of FABP5 in atherosclerotic plaque tissue was significantly higher than that in normal intima tissues [34]. The levels of this protein were observed to be significantly higher in PPD induration than in EC erythema, which could be potentially related to the formation of induration plaques and needs to be verified later. KEGG pathway analysis was then conducted for the DEPs. It was noted that compared with the PBS group, DEPs were mainly involved in the complement and coagulation cascades, the NOD-like receptor pathway, and the TNF pathway. Among them, the complement and coagulation grade pathway was the most represented, which could be related to DTH inflammation caused by the skin test reagents. In addition, a number of studies also confirmed that inflammation and coagulation are correlated, as inflammation can lead to coagulation activation, and coagulation can also significantly affect inflammatory activities [35]. Both the NOD-like receptor and TNF pathways can induce the expression of various proinflammatory factors. The PPAR signaling pathway, a distinct pathway between PPD induration and EC induration, is mainly involved in lipid metabolism and was not enhanced in immune-related pathways, which indicated to a certain extent that the main immune pathways of the DTH response were similar to those of EC erythema and PPD induration.

At present, due to the shortage of reagents in *guinea pigs* and the lack of suitable in vitro replacement indicators for induration and erythema, follow-up experiments are difficult to perform. However, our findings proved that the EC erythema and PPD induration reactions were similar as regards the aspects of skin test size, pathological level, infiltrating cell type, cytokines and DEPs. Overall, the data suggest that the generation of EC erythema has a certain diagnostic significance and provide a possible theoretical basis for the use of erythema as a diagnostic indicator of MTB infection.

## 4. Materials and Methods

### 4.1. Animals and Bacteria

Specific pathogen-free Hartley *guinea pigs* (female, 350–500 g) were obtained from the Institute for Laboratory Animal Resources of the China National Institute of Food and Drug Control (NIFDC) and were placed in an ABSL-2 laboratory. All the animals used in this study were treated in accordance with animal welfare standards and approved by the Laboratory Animal Welfare and Ethics Committee of the National Institutes for Food and Drug Control (NIFDC) (Protocol No. 2021(B)062).

For this study, cells of an MTB strain (American Type Culture Collection H37Ra strain) were harvested from a Lowenstein–Jensen medium culture, and the bacterial suspension was stored at −70 °C in our laboratory.

### 4.2. Sensitization of the Guinea Pigs 

The sensitization of the animals was carried out by subcutaneously injecting 0.5 mL of H37Ra bacterial solution into the groin; the dose used was 1 mg per animal. The sensitization scheme of the *guinea pigs* is depicted in Figure 1A. After 3 weeks, a second booster dose was injected, and the skin test was conducted 3 weeks after the last sensitization.

### 4.3. Skin Testing and Biopsy

The ventral hair of all *guinea pigs* was scraped, and the skin tests were then conducted, as shown in Figure 1A. Each *guinea pig* was injected intradermally with 0.1 mL of PPD (department preparation and preservation) and EC (Zhifei, Hefei, China), whereas PBS was used as a negative control. The PPD and EC skin test doses used were 0.1 µg and 1 µg, respectively. Induration or erythema was observed and measured 24 h after the injection and then expressed as the average value of transverse and vertical diameter measurements.

The *guinea pigs* were euthanized 24 h after the skin test, and the injection sites were disinfected with alcohol immediately. The skin tissues were removed, washed with PBS, and placed in a 10% formalin fixing solution for histological examination. The skin samples were processed for RNA extraction (16–20 mg skin sample in RNA later, Invitrogen, Carlsbad, CA, USA) or proteomic testing (1 µg skin sample in dry ice).

### 4.4. Pathological and Immunohistochemical Examination

Following formalin fixation, the tissues were embedded, sectioned (4 µm), stained with hematoxylin and eosin (H&E, Sigma-Aldrich, St. Louis, MO, USA), and examined under optical microscopy (Nikon, Tokyo, Japan). Vascular dilatation, infiltration of neutrophils, lymphocytes and mononuclear macrophages and edema in the dermal tissue were assessed semi-quantitatively. The scoring was: 0, no obvious lesions; 1, mild; 2, moderate; 3, severe; and 4, extremely severe.

The primary antibodies (anti-CD4, anti-CD8, Bio-Rad, Hercules, CA, USA) used for immunohistochemical staining were incubated with the tissues overnight at 4 °C. After washing with PBS, goat anti-mouse IgG (Servicebio, Wuhan, China) was incubated at 37 °C for 20 min. The sections were washed and stained with 3,3′-diaminobenzidine (DAB, Sigma-Aldrich). The slides were then observed under a microscope (SOPTOP, Ningbo, China), and the positive cells in the tissues were quantitatively analyzed using 3DHISTECH software v2.6 (3DHISTECH, Budapest, Hungary), which calculated the staining index (number of positive cells/total number of cells × 100%).

### 4.5. RT-PCR

The skin samples were homogenized, and RNA was extracted using the FastPure Cell/Tissue Total RNA Isolation Kit (Vazyme, Nanjing, China) based on the manufacturer’s protocol. The concentration and quality of the extracted RNA were estimated by a nucleic acid protein analyzer (Implen, Munich, Germany). The extracted RNA was reverse-transcribed into cDNA by employing the reverse transcription kit HiScript III RT SuperMix for qPCR (+gDNA wiper) (Vazyme), and the total amount of the template used was 1 µg. qPCR was carried out using primers for the *guinea pig* *β-actin*, *IFN-γ*, *TNF-α*, *IL-4*, *IL-6* and *CCL5* genes (Table 1), according to the Taq Pro Universal SYBR qPCR Master Mix kit’s instructions (Vazyme). Relative gene expression was calculated using the 2^−ΔΔCt^ method.

### 4.6. Protein Extraction and Trypsin Digestion

A total of four full-thickness skin samples from each group were used for protein extraction. The samples were crushed into powder form with liquid nitrogen, treated with lysis buffer and then ultrasonicated on ice for 2 min. After centrifugation (14,000× *g*, 20 min), the protein concentration in the supernatants was determined by the Bradford method [36].

The protein concentrations were adjusted to 0.5 µg/µL with 1×tetraethyl ammonium bromide (TEAB), and Tris (2-carboxyethyl) phosphine hydrochloride (TCEP) with a final concentration of 10 mM and 25 mM chloroacetamide (CAA, Sinopharm, Beijing, China) were added and reacted at 37 °C for 30 min. Beads, previously washed with water, were added, and the mixture was incubated at room temperature for 18 min. Subsequently, the magnetic beads were separated and cleaned, and 40 µL of 1 × TEAB was added to resuspend them. Trypsin (Promega, Madison, WI, USA) was added at a 1:10 trypsin-to-protein mass ratio, and incubation was performed at 37 °C for more than 4 h. Then, 5% trifluoroacetic acid (TFA, Sinopharm) was added to terminate the enzyme digestion, and the samples were freeze-dried.

### 4.7. Tandem Mass Tag (TMT) Labeling and Fractionation

The samples were labeled using the TMT-16plex Isobaric Label Reagent Set (Thermo Scientific, Waltham, MA, USA) according to the manufacturer’s instructions. Acetonitrile (41 µL, Sigma-Aldrich) was included in the thawed TMT reagent, and the mixture was shaken for 5 min and subjected to centrifugation. Thereafter, 100 µg of the digested samples were included for 1 h at room temperature, terminating the reaction with ammonia (Wako, Osaka, Japan). The samples were vortexed and freeze-dried under vacuum.

The samples were analyzed on a C18 liquid chromatography column (Waters, Milford, MA, USA), resulting in 10 fractions, which were then lyophilized.

### 4.8. LC-MS/MS Analysis

LC-MS/MS was performed using an L-3000 HPLC system (RIGOL, Beijing, China) and an ORBITRAP ECLIPSE mass spectrometer (Thermo Fisher). The freeze-dried sample was dissolved in 10 µL of mobile phase A (0.1% formic acid in 100% water), and the supernatant obtained after centrifugation of 1 µg sample was injected into a C18 liquid chromatography column. The separation flow rate was 600 nL/min, using mobile phase B (0.1% formic acid and 80% acetonitrile) for gradient elution. The isolated peptides were analyzed with a mass spectrometer equipped with FAIMS Pro™ Interface (Thermo Fisher), and the compensation voltage CV was switched every 1 s between ~45 and ~65. The ion source was Nanospray Flex™ (NSI, Thermo Fisher), the ion spray voltage was set to 2.0 kV, and the ion transport tube temperature was set to 320 °C. The mass spectra were obtained in data-dependent acquisition mode, the full scanning range of the mass spectra was *m*/*z* 350–1500, and the primary mass spectrum resolution was set to 120,000 (200 *m*/*z*). The automatic gain control (AGC) value was 100%, and the maximum C-trap injection time was 50 ms. Parent ions with the top 40 ionic intensity in the full scan were selected to be fragmented by high-energy collision dissociation (HCD), and secondary mass spectrometry detection was carried out. The secondary mass spectrometry resolution was set to 30,000 (200 *m*/*z*), the AGC was 100%, the maximum injection time was 54 ms, and the peptide fragment fragmentation collision energy was 36%.

### 4.9. Data Analysis

The database used was Uniprot_Cavia_porcellusfasta_25659_20220720. Raw data were processed using Proteome Discoverer v2.4.1.15 (Thermo Fisher) and annotated using the RefSeq protein database (24078 sequences, release 2017_03) with the SEQUEST algorithm. Trypsin was used as the cleavage enzyme, with a maximum of two missed cleavage sites allowed. We set a precursor mass tolerance of 20 ppm and a fragment mass tolerance of 0.05 Da. The false discovery rate (FDR) of proteins and peptide–spectrum matches (PSMs) was set to 0.01 [37]. Background correction was conducted for protein quantification, and then a median normalization was applied.

T-tests were used to identify proteins with significantly different expression. DEPs were identified using the FC > 1.2 and *p* < 0.05 criteria. Fisher’s exact tests were used to evaluate GO [38] and KEGG [39] enrichment of the DEPs.

### 4.10. Statistical Analysis

Data were analyzed using GraphPad Prism 9 (GraphPad Software, San Diego, CA, USA). T-tests and one- or two-way ANOVAs were used to assess differences between two or multiple groups. The results are shown as mean ± SEM. The differences were considered significant at * *p* < 0.05, ** *p* < 0.01, *** *p* < 0.001.

## Figures and Tables

**Table 1:** Primers used for qPCR.

Target	Primer Sequences
Cavia porcellus *β-actin*	Forward: 5′-CAGATGTGGATCAGCAAGCAGGAG-3′
	Reverse: 5′-CAAGAAAGGGTGTAACGCAGCAAAG-3′
Cavia porcellus *IFN-γ*	Forward: 5′-ATTTCGGTCAATGACGAGCAT-3′
	Reverse: 5′-GTTTCCTCTGGTTCGGTGACA-3′
Cavia porcellus *TNF-α*	Forward: 5′-CAAACCAGCAAGCAGAGGAGGAG-3′
	Reverse: 5′-TGAGGTACAGCCCATCCGAAGG-3′
Cavia porcellus *IL-4*	Forward: 5′-ACGGTCATTCTCTTCTGCCTCCTAG-3′
	Reverse: 5′-GAGAGTGTGTTGAGGTGCTGGATG-3′
Cavia porcellus *IL-6*	Forward: 5′-ACCCTGCTGGAGAAACTGGAGAC-3′
	Reverse: 5′-TTCTGCCTTCCCAATCTGCTTTCC-3′
Cavia porcellus *CCL5*	Forward: 5′-ACTCCTTGCTGCTTTGCCTACATC-3′
	Reverse: 5′-CCTGGCGGTTCTTTCGGGTAAC-3′

## Data Availability

Data are contained within the article and Supplementary Materials.

## Supplementary Materials

The supporting information can be downloaded at https://www.mdpi.com/article/10.3390/ijms242316612/s1.
