# Supplementary material for: A Comparative Study on the Mechanism of Delayed-Type Hypersensitivity Mediated by the Recombinant Mycobacterium tuberculosis Fusion Protein ESAT6-CFP10 and Purified Protein Derivative
Source: Int J Mol Sci. 2023 Nov 22;24(23):16612. doi: 10.3390/ijms242316612 (PMC10706316; doi:10.3390/ijms242316612)
Supplement: Supplementary file 1 [file ijms-24-16612-s001.zip › 222ijms-2697922-Supplementary File.pdf]

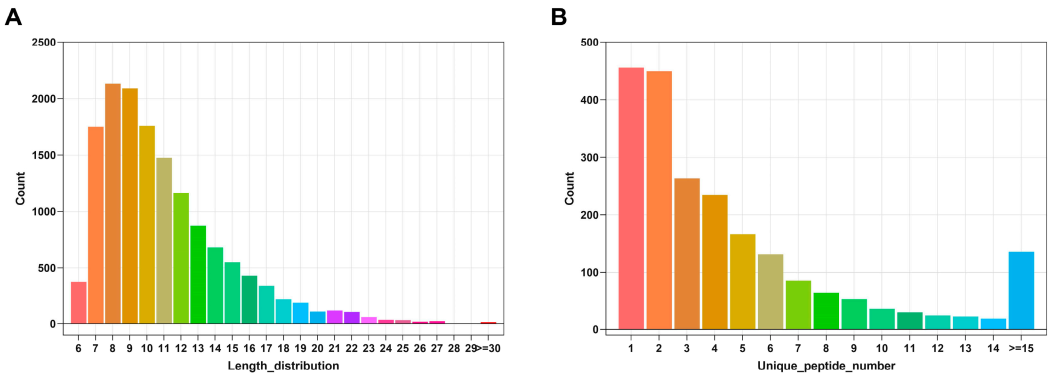

Supplementary Figure S1. Quality control of sample peptides. (A) The length distribution of peptide. The X-axis indicates the length of the identified peptide. The Y-axis represents the number of different peptides of the current length in the total number of peptides. (B) Number of unique peptide corresponding to the protein. The X-axis represents the number of unique peptides. The Y-axis indicates the number of the corresponding peptides.

Supplementary Table S1. DEPs in PPD induration vs. PBS.

Protein IDs	Gene names	Protein names	PPD induration average	PBS average	$\log_2$(FC)	p-value	Regulated Type
Q6WDN9	Alb	Preproalbumin	268996.470	162705.620	0.722	0.015	Up
H0UWJ0	LOC100721922	Uncharacterized protein	48865.980	24852.900	0.978	0.000	Up
P12387	C3	Complement C3 [Cleaved into: Complement C3 beta chain; C3-beta-c, C3bc; Complement C3 alpha chain; C3a anaphylatoxin; Complement C3b alpha' chain; Complement C3c alpha' chain fragment 1; Complement C3dg fragment; Complement C3g fragment; Complement C3d fragment; Complement C3f fragment; Complement C3c alpha' chain fragment 2]	33488.680	19210.780	0.799	0.009	Up
A0A286XNP9	FLNA	Filamin A	16343.750	12378.300	0.401	0.038	Up
Q71FK5	ACTB	Actin, cytoplasmic 1 (Beta-actin) [Cleaved into: Actin, cytoplasmic 1, N-terminally processed]	13081.300	9844.230	0.411	0.026	Up
Q60486		Alpha-macroglobulin	18913.200	9050.280	1.064	0.000	Up
H0UXJ	HSP9	Heat shock protein 90 alpha family	15382.220	11515.170	0.422	0.030	Up

8	0AA1	class A member 1					
H0VJK 2	PLG	Plasminogen, EC 3.4.21.7	9810.880	5604.650	0.807	0.008	Up
A0A28 6XA82	LOC1 00728 345	Anaphylatoxin-like domain-containing protein	10361.770	5883.500	0.816	0.003	Up
H0VTA 4	GC	Vitamin D-binding protein (Gc-globulin) (Group-specific component)	19640.900	10395.200	0.918	0.007	Up
A0A28 6XB48	FN1	Fibronectin	5332.020	3852.900	0.465	0.038	Up
H0V1 W9	HSP9 0AB1	Heat shock protein 90 alpha family class B member 1	6144.850	4556.880	0.433	0.023	Up
H0VM5 9	KNG 1	Kininogen 1	8342.030	5058.820	0.722	0.014	Up
Q60488		Murinoglobulin	10965.350	5713.820	0.941	0.003	Up
P01862		Ig gamma-2 chain C region	24104.850	13374.900	0.848	0.002	Up
H0V65 7		Uncharacterized protein	7816.700	4831.380	0.696	0.018	Up
A0A7T 7JJ63		Hemoglobin subunit beta	13640.900	9558.770	0.516	0.026	Up
H0W2 U2	HRG	Histidine rich glycoprotein	16291.470	9171.750	0.832	0.029	Up
A0A28 6Y5M0	ITIH4	Inter-alpha-trypsin inhibitor heavy chain 4	6383.650	3241.530	0.978	0.004	Up
H0UW E7	GLN A1	Globin A1	570.400	407.000	0.485	0.046	Up
H0VLV 4	LAP3	Cytosol aminopeptidase, EC 3.4.11.1, EC 3.4.11.5, EC 3.4.13.23 (Cysteinylglycine-S-conjugate dipeptidase) (Leucine aminopeptidase 3) (Leucyl aminopeptidase) (Proline aminopeptidase) (Prolyl aminopeptidase)	6124.020	4241.000	0.526	0.045	Up
A0A28 6XP66	YWH AG	Tyrosine 3-monooxygenase/tryptophan 5-monooxygenase activation protein gamma	7745.320	6158.950	0.333	0.042	Up
H0V4A 3	APO B	Apolipoprotein B	4641.120	3091.600	0.585	0.006	Up
H0V9B 7	MSN	Moesin	8181.170	6173.750	0.411	0.037	Up
A0A28	LOC1	Uncharacterized protein	2393.200	1607.600	0.575	0.007	Up

6XED8	00721922						
A0A286XCE4	CP	Ceruloplasmin	5427.000	3061.320	0.824	0.006	Up
H0VBA9	CAPG	Macrophage-capping protein (Actin regulatory protein CAP-G)	3324.800	2505.820	0.411	0.043	Up
A0A286XCX2	MYH11	Myosin heavy chain 11	2278.400	1665.670	0.454	0.032	Up
A0A286XGL1	YWHAB	Tyrosine 3-monooxygenase/tryptophan 5-monooxygenase activation protein beta	1312.930	954.370	0.465	0.036	Up
H0VT62	Serpina3k	SERPIN domain-containing protein	4369.700	1974.450	1.144	0.005	Up
H0VSD9	GBP1	Guanylate binding protein 1	5271.900	2414.530	1.124	0.044	Up
H0WD53	SERPINA3	SERPIN domain-containing protein	6204.430	3247.400	0.934	0.004	Up
H0UXD2	LCP1	Lymphocyte cytosolic protein 1	4037.550	2301.270	0.807	0.007	Up
O70159	AHSG	Alpha-2-HS-glycoprotein (Fetuin-A)	5151.550	2209.250	1.220	0.025	Up
A0A222AH49	HBA	Hemoglobine alpha globin subunit	13253.020	8853.030	0.585	0.006	Up
H0VDI5	HPX	Hemopexin	4173.980	2402.380	0.799	0.032	Up
H0V0Z9	FGA	Fibrinogen alpha chain	4689.800	3126.230	0.585	0.015	Up
H0V2R8	WARS1	Tryptophan--tRNA ligase, cytoplasmic, EC 6.1.1.2 (Tryptophanyl-tRNA synthetase)	5572.500	2847.200	0.971	0.041	Up
A0A286Y0Y6	SFN	Stratifin	5103.130	2808.600	0.864	0.047	Up
H0VLZ0	STAT1	Signal transducer and activator of transcription	3118.300	1761.250	0.824	0.009	Up
H0VD80	FGB	Fibrinogen beta chain	4919.250	3741.400	0.390	0.041	Up
H0V2M6		Uncharacterized protein	3184.250	1919.050	0.731	0.029	Up
H0VUK8		Complement factor B, EC 3.4.21.47 (C3/C5 convertase)	4769.730	2765.750	0.782	0.023	Up
P22325		Alpha-1-antiproteinase S, APS (Alpha-1-antitrypsin) (Alpha-1-proteinase inhibitor)	9099.180	4690.070	0.956	0.012	Up

H0UTK8	ITIH1	Inter-alpha-trypsin inhibitor heavy chain 1	4440.320	2661.080	0.740	0.022	Up
A0A286XVI4	CORO1A	Coronin	3443.070	2020.950	0.766	0.010	Up
H0VZ24	PGD	6-phosphogluconate dehydrogenase, decarboxylating, EC 1.1.1.44	3026.380	2274.100	0.411	0.039	Up
H0W7H0	TAGLN2	Transgelin	2855.950	1884.030	0.604	0.015	Up
H0VH18	ITIH2	Inter-alpha-trypsin inhibitor heavy chain 2	3064.280	1790.920	0.774	0.011	Up
H0UY41	C5	Complement C5	2427.480	1575.620	0.623	0.006	Up
A9QUC5	FABP5	E-FABP (Fatty acid binding protein 5)	2825.200	1903.100	0.566	0.010	Up
H0VXN4		Peptidyl-prolyl cis-trans isomerase, PPIase, EC 5.2.1.8	6342.850	4401.850	0.526	0.025	Up
Q9Z201		Fibrinogen A-alpha chain	93.300	49.900	0.903	0.008	Up
H0UXA8	TALDO1	Transaldolase, EC 2.2.1.2	4078.780	3085.800	0.401	0.049	Up
Q9WTT1		Antithrombin-III (Serpin C1)	6305.920	3282.330	0.941	0.010	Up
A0A286XVP4	PSMA6	Proteasome subunit alpha type	2849.000	2177.020	0.390	0.034	Up
H0W7R3	CLIC1	Chloride intracellular channel protein	1969.780	1411.280	0.485	0.028	Up
A0A286XW34	Pfn1	Profilin	4408.920	3088.530	0.516	0.029	Up
A0A286XZC3		Ig-like domain-containing protein	925.280	564.250	0.714	0.002	Up
J7NP09	IFGGC1	Interferon-gamma-inducible GTPase IFGGC1 protein	811.650	459.500	0.824	0.026	Up
H0V674	Cap1	Adenylyl cyclase-associated protein	1873.800	1405.070	0.411	0.029	Up
H0VIN5	FETUB	Fetuin B	2572.350	1423.250	0.856	0.012	Up
A0A286XX92		Adenylyl cyclase-associated protein	239.700	167.750	0.516	0.023	Up
H0VQ28	PPA1	Inorganic diphosphatase, EC 3.6.1.1	1927.400	1236.530	0.642	0.012	Up
H0VMF5	CA1	Carbonic anhydrase, EC 4.2.1.1	1698.430	1092.150	0.642	0.016	Up
H0W8M5		Uncharacterized protein	1532.670	1058.980	0.536	0.033	Up

H0VL35	APOH	Beta-2-glycoprotein 1 (Apolipoprotein H) (Beta-2-glycoprotein I)	1701.170	1125.000	0.595	0.014	Up
H0V0F4		Uncharacterized protein	1946.200	1306.420	0.575	0.049	Up
A0A286XTI6	MAT2A	S-adenosylmethionine synthase, EC 2.5.1.6	1081.200	840.000	0.367	0.039	Up
Q99JA1	C4BPA	C4bp alpha-chain	1250.400	651.150	0.941	0.001	Up
A0A286XX81	CSRP1	Cysteine and glycine rich protein 1	1348.570	1041.170	0.379	0.015	Up
H0W0E9	HNRNPF	Heterogeneous nuclear ribonucleoprotein F	964.820	700.400	0.465	0.033	Up
H0W7A0		NTR domain-containing protein	1649.630	978.420	0.757	0.003	Up
A0A286X9V2		Ig-like domain-containing protein	3224.550	1789.650	0.848	0.001	Up
A0A077SA01	LYZF4	Lysozyme, EC 3.2.1.17 (1,4-beta-N-acetylmuramidase C)	2575.180	1552.470	0.731	0.025	Up
A0A286XGB6	GLUL	Glutamine synthetase, EC 6.3.1.2	1849.830	1284.150	0.526	0.046	Up
A0A286Y4C5	C8A	Complement C8 alpha chain	769.920	496.500	0.632	0.004	Up
H0VA29	ALAD	Delta-aminolevulinic acid dehydratase, EC 4.2.1.24	560.050	377.950	0.566	0.003	Up
H0UXF4	GPLD1	Phosphatidylinositol-glycan-specific phospholipase D, EC 3.1.4.50 (Glycosyl-phosphatidylinositol-specific phospholipase D)	1576.900	1007.120	0.651	0.043	Up
A0A286XA60	LOC100713681	Asparagine synthetase [glutamine-hydrolyzing], EC 6.3.5.4	187.300	144.330	0.379	0.013	Up
H0WDA4	ARPC1B	Actin-related protein 2/3 complex subunit	1138.120	847.200	0.422	0.048	Up
H0VZS4	F2	Prothrombin, EC 3.4.21.5 (Coagulation factor II)	728.120	567.120	0.356	0.028	Up
A0A286XWN4	CSTB	Cystatin B	903.330	648.170	0.475	0.031	Up
A0A286XN02	LOC100714630	GB1/RHD3-type G domain-containing protein	1646.320	956.120	0.782	0.024	Up
A0A286XW44	ARPC2	Arp2/3 complex 34 kDa subunit	1562.970	1193.500	0.390	0.034	Up
H0VH3	ERP2	Endoplasmic reticulum resident	781.550	502.400	0.642	0.012	Up

3	9	protein 29					
A0A286XCQ3	PSME1	Proteasome activator subunit 1	1285.620	885.680	0.536	0.014	Up
H0VGZ9	AMBP	Protein AMBP	1411.650	880.080	0.678	0.010	Up
H0V4A7	KLKB1	Kallikrein B1	1450.150	911.330	0.669	0.014	Up
H0VZH7	TNC	Tenascin C	716.170	405.000	0.824	0.007	Up
H0VLB4	ALDOB	Fructose-bisphosphate aldolase, EC 4.1.2.13	520.880	354.250	0.556	0.043	Up
A0A286X772	AGT	Angiotensinogen (Serpin A8)	545.800	302.300	0.856	0.002	Up
H0VTW7	C7	Complement C7	783.920	542.380	0.536	0.027	Up
H0W6R6		Ig-like domain-containing protein	2004.220	1049.200	0.934	0.008	Up
A0A286XLN5	TAGLN	Transgelin	1979.220	1196.120	0.722	0.017	Up
A0A286Y2V7	COTL1	Coactosin like F-actin binding protein 1	2007.200	1235.750	0.696	0.012	Up
H0VGU4	NUDC	Nuclear migration protein nudC (Nuclear distribution protein C homolog)	959.170	696.070	0.465	0.034	Up
H0VI24		SERPIN domain-containing protein	1257.580	960.730	0.390	0.047	Up
H0W4Y5	EFHD2	EF-hand domain family member D2	967.820	653.380	0.566	0.007	Up
A0A286XJ60	SERPINF2	Serpin family F member 2	988.000	483.450	1.029	0.001	Up
H0W3K9	CLEC3B	C-type lectin domain family 3 member B	1376.680	1039.850	0.401	0.042	Up
H0VQN7		Uncharacterized protein	1236.750	704.700	0.816	0.037	Up
A0A286Y4M0	CAPRIN1	Cell cycle associated protein 1	1011.020	731.450	0.465	0.010	Up
H0VXU5	IFGGC3	Interferon-gamma-inducible GTPase IFGGC3 protein	427.250	266.580	0.678	0.032	Up
A0A286XP53	TBCA	Tubulin-specific chaperone A	748.000	584.480	0.356	0.027	Up
H0VMD5	TTR	Transthyretin	1216.500	539.830	1.170	0.028	Up
A0A286Y257	PSME2	Proteasome activator subunit 2	962.620	597.430	0.687	0.010	Up

H0W8T6	SERPINA6	Serpin family A member 6	2121.600	1224.880	0.791	0.030	Up
A0A286XNN0	PTPN6	Tyrosine-protein phosphatase non-receptor type, EC 3.1.3.48	845.550	557.700	0.604	0.028	Up
H0VM65	SERPINB1	Serpin family B member 1	422.300	309.250	0.454	0.032	Up
A0A7T8G3K2		MHC class I antigen	680.620	431.430	0.660	0.003	Up
A0A286XJR4	MANF	Mesencephalic astrocyte derived neurotrophic factor	713.900	511.020	0.485	0.035	Up
H0VVP2	CLU	Clusterin	230.850	155.350	0.575	0.015	Up
A0A286XL53	FBLN1	Fibulin-1	692.880	483.950	0.516	0.041	Up
H0UU40	LIMA1	LIM domain and actin binding 1	962.750	662.020	0.536	0.026	Up
A0A286XFJ4		Uncharacterized protein	861.000	561.500	0.614	0.010	Up
A0A0D9QUG2	APOA1	Apolipoprotein A-I	518.550	366.920	0.496	0.020	Up
H0VKX9	C8B	Complement component C8 beta chain (Complement component 8 subunit beta)	306.900	210.350	0.546	0.001	Up
Q04962	F12	Coagulation factor XII, EC 3.4.21.38 (Hageman factor, HAF) [Cleaved into: Coagulation factor XIIa heavy chain; Coagulation factor XIIa light chain]	1674.400	900.970	0.895	0.026	Up
H0UTL6	ARHGDIB	Rho GDP dissociation inhibitor beta	1431.650	738.450	0.956	0.001	Up
A0A286XYF7	C6	Complement component C6	112.030	77.580	0.526	0.017	Up
H0W145	ISG15	ISG15 ubiquitin like modifier	357.200	154.120	1.214	0.004	Up
H0VEH6	LOC100714630	GB1/RHD3-type G domain-containing protein	708.720	429.730	0.722	0.048	Up
H0WDA3	ARPC4	Actin-related protein 2/3 complex subunit 4	596.730	463.200	0.367	0.046	Up
A0A286X9U8	RPL14	60S ribosomal protein L14	393.880	306.880	0.356	0.002	Up
H0WE16	UPP1	Uridine phosphorylase, EC 2.4.2.3	757.380	420.320	0.848	0.004	Up
A0A28		Uncharacterized protein	1592.830	1062.050	0.585	0.039	Up

6X971							
H0UTX 4	Rps12	40S ribosomal protein S12	300.020	236.480	0.345	0.027	Up
H0UU6 3	GMF G	Glia maturation factor	70.580	48.020	0.556	0.032	Up
H0W5 Q4	PGLY RP2	Peptidoglycan recognition protein 2	791.900	415.600	0.934	0.006	Up
H0V9N 6	CFP	Properdin	557.620	358.000	0.642	0.010	Up
H0V12 9	PLEK	Pleckstrin	495.900	234.320	1.084	0.026	Up
B0FJL6	TPT1	TPT1	615.800	430.880	0.516	0.038	Up
A0A28 6XIK7	SRSF 3	Serine and arginine rich splicing factor 3	1228.830	857.500	0.516	0.039	Up
H0UX U7	LOC1 00714 902	GB1/RHD3-type G domain-containing protein	242.550	109.850	1.144	0.004	Up
A0A28 6XBE2	PAK2	Non-specific serine/threonine protein kinase, EC 2.7.11.1	246.820	180.580	0.454	0.048	Up
A0A28 6XUG1	RAN BP1	RAN binding protein 1	302.170	204.400	0.566	0.002	Up
H0W2B 0		Serpin family G member 1	407.450	256.750	0.669	0.007	Up
A0A28 6XZW3	GMF B	Glia maturation factor	444.350	270.950	0.714	0.034	Up
H0VRD 6	SF3A 1	Splicing factor 3a subunit 1	299.930	219.570	0.454	0.037	Up
H0VK W6	EIF2 AK2	Eukaryotic translation initiation factor 2 alpha kinase 2 (Interferon-induced double-stranded RNA-activated protein kinase)	451.950	307.770	0.556	0.034	Up
H0VZY 3	F9	Coagulation factor IX, EC 3.4.21.22 (Christmas factor)	160.220	104.070	0.623	0.038	Up
H0VQE 8	NAA 50	N(alpha)-acetyltransferase 50, NatE catalytic subunit	444.780	350.230	0.345	0.045	Up
H0VM C7	IST1	IST1 homolog (Charged multivesicular body protein 8)	471.620	345.700	0.444	0.037	Up
A0A28 6X869	PTX3	Pentraxin 3	435.800	290.930	0.585	0.034	Up
A0A28 6Y431	ATP5 IF1	ATP synthase F1 subunit epsilon (ATPase inhibitor, mitochondrial)	1215.330	988.400	0.299	0.009	Up
H0UXT 0	PSMB 9	Proteasome subunit beta	116.480	83.080	0.485	0.027	Up
A0A28	NMI	N-myc and STAT interactor	122.030	52.980	1.202	0.005	Up

6XWP2							
H0WCJ7	MYL10	Myosin light chain 10	20.100	14.430	0.475	0.041	Up
A0A286Y2K1		Uncharacterized protein	737.950	443.650	0.731	0.046	Up
H0VDE9	C1S	Complement C1s	199.350	132.150	0.595	0.048	Up
A0A286XQW9	FNBP1	Formin binding protein 1	364.700	258.700	0.496	0.025	Up
H0V7P7	HCLS1	Hematopoietic cell-specific Lyn substrate 1	219.480	167.750	0.390	0.004	Up
A0A286Y252	SH3BGRL3	SH3 domain binding glutamate rich protein like 3	883.070	550.580	0.678	0.028	Up
A0A286XW79	Tpd52	Tumor protein D52	102.500	64.620	0.669	0.007	Up
P49255	PTX2	Serum amyloid P-component, SAP	608.270	371.720	0.714	0.000	Up
H0UVX5	PML	Promyelocytic leukemia	44.300	31.670	0.485	0.007	Up
H0UTB5	PAK1	Non-specific serine/threonine protein kinase, EC 2.7.11.1	321.950	238.930	0.433	0.048	Up
A0A286XHI2		COesterase domain-containing protein	704.620	350.200	1.007	0.006	Up
H0VPX9		Haptoglobin	853.220	309.320	1.465	0.026	Up
A0A286XH52	CNN2	Calponin	229.820	145.400	0.660	0.009	Up
H0VGX4	BZW1	Basic leucine zipper and W2 domains 1	341.470	267.020	0.356	0.019	Up
A0A286XXT2	C1QA	Complement C1q subcomponent subunit A	204.270	130.400	0.651	0.007	Up
H0W265	CTSZ	Cathepsin X, EC 3.4.18.1	228.570	167.000	0.454	0.036	Up
H0V0A8	PLS1	Plastin 1	278.220	186.730	0.575	0.014	Up
H0W2Q0	ANP32B	Acidic nuclear phosphoprotein 32 family member B	977.900	586.630	0.740	0.042	Up
H0V391	SNRNP40	Small nuclear ribonucleoprotein U5 subunit 40	230.050	166.980	0.465	0.008	Up
H0V9C6	HMGCS1	Hydroxymethylglutaryl-CoA synthase, HMG-CoA synthase, EC 2.3.3.10 (3-hydroxy-3-methylglutaryl coenzyme A synthase)	345.700	267.320	0.367	0.023	Up
H0UTI	SNX1	Sorting nexin-1	99.500	66.200	0.585	0.026	Up

6							
A0A28 6XZL9	AZG P1	Alpha-2-glycoprotein 1, zinc-binding	468.120	310.880	0.595	0.007	Up
A0A28 6XFH5	APEX 1	DNA-(apurinic or apyrimidinic site) endonuclease, EC 3.1.-.-	226.120	147.470	0.614	0.043	Up
A0A28 6XSX9	MAR CKSL 1	MARCKS like 1	238.320	162.200	0.556	0.024	Up
H0VJV 9	RPL2 9	60S ribosomal protein L29	615.880	446.420	0.465	0.040	Up
H0V45 6	PSTPI P2	Proline-serine-threonine phosphatase interacting protein 2	72.000	51.750	0.475	0.041	Up
H0UX K2	CTSH	Cathepsin H	246.780	184.230	0.422	0.025	Up
A0A28 6XTY6	ARRB 1	Arrestin beta 1	154.550	122.430	0.333	0.001	Up
H0W33 3	LGA LS3B P	Galectin 3 binding protein	321.900	180.550	0.832	0.006	Up
A0A28 6XXB1	DNM 1L	Dynamin-1-like protein, EC 3.6.5.5	386.350	294.350	0.390	0.043	Up
H0UZJ 6	PDLI M1	PDZ and LIM domain 1	281.500	166.400	0.757	0.015	Up
H0UV Q4	LGM N	Legumain, EC 3.4.22.34 (Asparaginyl endopeptidase) (Protease, cysteine 1)	420.230	305.620	0.465	0.034	Up
H0VBL 8	C1QB	Adiponectin A (Complement C1q B chain)	250.220	155.550	0.687	0.002	Up
H0UZ G7	TMO D3	Tropomodulin 3	340.550	236.620	0.526	0.025	Up
Q07E14	TES	Testin	225.700	170.180	0.411	0.035	Up
H0VTV 3		Uncharacterized protein	222.800	117.730	0.918	0.020	Up
H0VA V9	CD44	CD44 antigen (Extracellular matrix receptor III) (GP90 lymphocyte homing/adhesion receptor) (HUTCH-I) (Hermes antigen) (Hyaluronate receptor) (Phagocytic glycoprotein 1) (Phagocytic glycoprotein I)	154.500	111.650	0.465	0.013	Up
H0UZ N4	FER MT3	Fermitin family member 3	232.980	192.120	0.275	0.035	Up
H0UXT 9	RBP4	Apolipoprotein D, Apo-D	315.620	183.500	0.782	0.003	Up

A0A286Y3F1	GDA	Guanine deaminase, Guanase, EC 3.5.4.3 (Guanine aminohydrolase)	70.750	53.550	0.401	0.035	Up
H0VKC8	FGL2	Fibrinogen like 2	349.450	260.350	0.422	0.043	Up
H0V077		Vitronectin	437.750	175.730	1.316	0.015	Up
H0VF83	LOC100718647	Uncharacterized protein	201.780	112.920	0.840	0.018	Up
H0VA43	ERO1A	Endoplasmic reticulum oxidoreductase 1 alpha	102.220	82.270	0.310	0.045	Up
A0A286X7T0	PUF60	Poly(U)-binding-splicing factor PUF60 (60 kDa poly(U)-binding-splicing factor)	139.220	98.880	0.496	0.010	Up
A0A286XRG0	UBL4A	Ubiquitin-like protein 4A	274.320	192.080	0.516	0.043	Up
H0VPR6	FCN1	Ficolin 1	269.250	147.280	0.872	0.004	Up
H0V4Y6	CHI3L1	Chitinase-3-like protein 1	117.180	79.580	0.556	0.027	Up
H0VV85	CASP10	Caspase 10	77.100	54.800	0.496	0.003	Up
H0WC19	Set	Uncharacterized protein	306.020	154.350	0.986	0.023	Up
A0A286XUI5	CALU	Calumenin	296.750	169.200	0.807	0.024	Up
A0A286XTN8	PPP1R9B	Protein phosphatase 1 regulatory subunit 9B	95.930	65.250	0.556	0.049	Up
P22032	MBP1	Eosinophil granule major basic protein 1, MBP-1	131.430	96.570	0.444	0.002	Up
H0VNM7	LNPEP	Leucyl and cystinyl aminopeptidase	63.750	49.600	0.367	0.008	Up
H0W255		Uncharacterized protein	144.180	103.270	0.485	0.009	Up
A0A286XCT1	CXCL10	C-X-C motif chemokine ligand 10	205.320	106.050	0.956	0.004	Up
H0VI18	TNFAIP2	TNF alpha induced protein 2	42.950	29.520	0.536	0.029	Up
A0A286XI21		Translation machinery-associated protein 7 (Coiled-coil domain-containing protein 72)	235.820	171.100	0.465	0.042	Up
A0A286Y1R9	ACOT7	Acyl-CoA thioesterase 7	149.120	112.300	0.411	0.010	Up
A0A28	PSAT	Phosphoserine aminotransferase,	457.280	343.250	0.411	0.038	Up

6XN71	1	EC 2.6.1.52					
A0A28 6Y400		F-box protein 6	167.350	97.600	0.774	0.037	Up
A0A28 6XES5	IGFA LS	Insulin like growth factor binding protein acid labile subunit	58.380	29.620	0.978	0.007	Up
A0A28 6Y071	UBE2 I	Ubiquitin conjugating enzyme E2 I	189.930	144.600	0.390	0.015	Up
A0A28 6XCI7	UBE2 E2	Ubiquitin conjugating enzyme E2 E2	124.000	93.200	0.411	0.015	Up
A0A28 6XK05		Serum amyloid A protein	166.970	70.170	1.251	0.022	Up
H0VRD 0	CAST OR1	Cytosolic arginine sensor for mTORC1 subunit 1 (GATS-like protein 3)	119.930	91.380	0.390	0.047	Up
H0VZC 4	LRG1	Leucine rich alpha-2-glycoprotein 1	193.400	138.150	0.485	0.026	Up
H0UU M2	LOC1 00731 677	Lysozyme, EC 3.2.1.17 (1,4-beta-N-acetylmuramidase C)	190.300	102.200	0.895	0.043	Up
H0UYR 4	HPRT 1	Hypoxanthine phosphoribosyltransferase, EC 2.4.2.8	234.180	160.880	0.546	0.013	Up
H0VLI 4	AFP	Alpha fetoprotein	777.250	455.000	0.774	0.007	Up
H0VS8 6	WDR 91	WD repeat-containing protein 91	187.300	145.300	0.367	0.047	Up
H0VC M4	SNRP C	U1 small nuclear ribonucleoprotein C, U1 snRNP C, U1-C, U1C	200.650	159.570	0.333	0.040	Up
H0V1F 9	MCM 4	DNA replication licensing factor MCM4, EC 3.6.4.12	96.800	78.150	0.310	0.049	Up
A0A28 6XGM3	RAD2 3B	UV excision repair protein RAD23	240.880	143.150	0.748	0.031	Up
A0A28 6XC44	STM N1	Stathmin	344.170	183.000	0.911	0.027	Up
H0WE2 4	PARP 14	Poly [ADP-ribose] polymerase, PARP, EC 2.4.2.-	167.680	106.930	0.651	0.018	Up
H0VQ Z5	VPS4 B	Vesicle-fusing ATPase, EC 3.6.4.6	217.520	160.550	0.433	0.044	Up
A0A28 6XDX4		Ig-like domain-containing protein	244.930	182.300	0.422	0.048	Up
H0VBD 9	SF3B6	Splicing factor 3b subunit 6	255.520	174.470	0.546	0.014	Up
H0V04 9		Uncharacterized protein	452.900	238.720	0.926	0.009	Up

A0A286XS98		LIM zinc-binding domain-containing protein	128.250	70.950	0.856	0.001	Up
A0A286Y4S2	LOC100722668	Uncharacterized protein	146.000	74.670	0.971	0.004	Up

Supplementary Table S2. DEPs in EC erythema vs. PBS.

**Protein IDs**	**Gene names**	**Protein names**	**EC erythema average**	**PBS average**	**log$_2$(FC)**	**p-value**	**Regulated Type**
Q6WDN9	Alb	Preproalbumin	262200.680	162705.620	0.687	0.009	Up
H0UWJ0	LOC100721922	Uncharacterized protein	40470.350	24852.900	0.705	0.012	Up
P12387	C3	Complement C3 [Cleaved into: Complement C3 beta chain; C3-beta-c, C3bc; Complement C3 alpha chain; C3a anaphylatoxin; Complement C3b alpha' chain; Complement C3c alpha' chain fragment 1; Complement C3dg fragment; Complement C3g fragment; Complement C3d fragment; Complement C3f fragment; Complement C3c alpha' chain fragment 2]	32813.570	19210.780	0.774	0.018	Up
Q60486		Alpha-macroglobulin	16997.550	9050.280	0.911	0.007	Up
H0VJK2	PLG	Plasminogen, EC 3.4.21.7	10385.750	5604.650	0.888	0.018	Up
A0A286XA82	LOC100728345	Anaphylatoxin-like domain-containing protein	9961.920	5883.500	0.757	0.013	Up
H0VTA4	GC	Vitamin D-binding protein (Gc-globulin) (Group-specific component)	18373.980	10395.200	0.824	0.011	Up
H0VM59	KNG1	Kininogen 1	8063.150	5058.820	0.669	0.020	Up
Q60488		Murinoglobulin	9757.830	5713.820	0.774	0.009	Up
P01862		Ig gamma-2 chain C region	21207.200	13374.900	0.669	0.021	Up
H0W2U2	HRG	Histidine rich glycoprotein	15937.700	9171.750	0.799	0.011	Up
A0A286Y5M0	ITIH4	Inter-alpha-trypsin inhibitor heavy chain 4	6029.080	3241.530	0.895	0.005	Up

H0VLV4	LAP3	Cytosol aminopeptidase, EC 3.4.11.1, EC 3.4.11.5, EC 3.4.13.23 (Cysteinylglycine-S-conjugate dipeptidase) (Leucine aminopeptidase 3) (Leucyl aminopeptidase) (Proline aminopeptidase) (Prolyl aminopeptidase)	6037.100	4241.000	0.506	0.029	Up
A0A286XED8	LOC100721922	Uncharacterized protein	2142.380	1607.600	0.411	0.010	Up
A0A286XCE4	CP	Ceruloplasmin	4928.500	3061.320	0.687	0.011	Up
H0VT62	Serpina3k	SERPIN domain-containing protein	3297.530	1974.450	0.740	0.011	Up
H0VSD9	GBP1	Guanylate binding protein 1	6919.550	2414.530	1.521	0.002	Up
H0WD53	SERPINA3	SERPIN domain-containing protein	5271.820	3247.400	0.696	0.012	Up
H0UXD2	LCP1	Lymphocyte cytosolic protein 1	4113.900	2301.270	0.840	0.012	Up
O70159	AHSG	Alpha-2-HS-glycoprotein (Fetuin-A)	3475.970	2209.250	0.651	0.031	Up
H0VDI5	HPX	Hemopexin	4161.950	2402.380	0.791	0.002	Up
H0V2R8	WARS1	Tryptophan--tRNA ligase, cytoplasmic, EC 6.1.1.2 (Tryptophanyl-tRNA synthetase)	6890.000	2847.200	1.275	0.002	Up
H0VLZ0	STAT1	Signal transducer and activator of transcription	2994.900	1761.250	0.766	0.007	Up
H0V2M6		Uncharacterized protein	3341.980	1919.050	0.799	0.020	Up
H0VUK8		Complement factor B, EC 3.4.21.47 (C3/C5 convertase)	4743.730	2765.750	0.782	0.022	Up
P22325		Alpha-1-antiproteinase S, APS (Alpha-1-antitrypsin) (Alpha-1-proteinase inhibitor)	7458.100	4690.070	0.669	0.007	Up
H0UTK8	ITIH1	Inter-alpha-trypsin inhibitor heavy chain 1	4173.550	2661.080	0.651	0.022	Up
A0A286XVI4	CORO1A	Coronin	3927.350	2020.950	0.956	0.008	Up
H0VZ24	PGD	6-phosphogluconate dehydrogenase, decarboxylating, EC 1.1.1.44	3125.700	2274.100	0.454	0.027	Up

H0VH18	ITIH2	Inter-alpha-trypsin inhibitor heavy chain 2	2905.250	1790.920	0.696	0.014	Up
A0A286XJN1	IDO1	Indoleamine 2,3-dioxygenase 1	6419.400	2385.530	1.428	0.004	Up
H0UY41	C5	Complement C5	2289.850	1575.620	0.536	0.030	Up
A0A286Y4G4	KRT3	Keratin 3	527.600	775.470	-0.556	0.019	Down
Q9WTT1		Antithrombin-III (Serpin C1)	5494.150	3282.330	0.740	0.019	Up
J7NP09	IFGGC1	Interferon-gamma-inducible GTPase IFGGC1 protein	973.200	459.500	1.084	0.002	Up
H0VIN5	FETUB	Fetuin B	2285.180	1423.250	0.687	0.010	Up
H0VQ28	PPA1	Inorganic diphosphatase, EC 3.6.1.1	1861.750	1236.530	0.595	0.033	Up
H0VL35	APOH	Beta-2-glycoprotein 1 (Apolipoprotein H) (Beta-2-glycoprotein I)	1784.600	1125.000	0.669	0.007	Up
Q99JA1	C4BPA	C4bp alpha-chain	1113.620	651.150	0.774	0.005	Up
H0W7A0		NTR domain-containing protein	1600.970	978.420	0.714	0.012	Up
A0A077SA01	LYZF4	Lysozyme, EC 3.2.1.17 (1,4-beta-N-acetylmuramidase C)	2582.970	1552.470	0.731	0.003	Up
A0A286XGB6	GLUL	Glutamine synthetase, EC 6.3.1.2	1885.120	1284.150	0.556	0.013	Up
A0A286Y4C5	C8A	Complement C8 alpha chain	744.030	496.500	0.585	0.020	Up
H0UXF4	GPLD1	Phosphatidylinositol-glycan-specific phospholipase D, EC 3.1.4.50 (Glycosyl-phosphatidylinositol-specific phospholipase D)	1441.100	1007.120	0.516	0.024	Up
A0A286XA60	LOC100713681	Asparagine synthetase [glutamine-hydrolyzing], EC 6.3.5.4	179.350	144.330	0.310	0.047	Up
H0V150	HMGB2	High mobility group box 2	749.300	496.500	0.595	0.043	Up
A0A286XN02	LOC100714630	GB1/RHD3-type G domain-containing protein	1831.230	956.120	0.941	0.004	Up
A0A286XCQ3	PSME1	Proteasome activator subunit 1	1190.720	885.680	0.422	0.045	Up
H0VGZ	AMB	Protein AMBP	1275.970	880.080	0.536	0.029	Up

9	P						
H0V4A7	KLKB1	Kallikrein B1	1369.600	911.330	0.585	0.028	Up
A0A286X7T6	SOD2	Superoxide dismutase, EC 1.15.1.1	2786.500	1527.580	0.864	0.003	Up
H0VLB4	ALDOB	Fructose-bisphosphate aldolase, EC 4.1.2.13	481.450	354.250	0.444	0.041	Up
A0A286X772	AGT	Angiotensinogen (Serpin A8)	523.000	302.300	0.791	0.003	Up
A0A286Y2V7	COTL1	Coactosin like F-actin binding protein 1	2047.150	1235.750	0.731	0.022	Up
A0A286XJ60	SERPINF2	Serpin family F member 2	835.380	483.450	0.791	0.012	Up
H0VQN7		Uncharacterized protein	1276.500	704.700	0.856	0.005	Up
H0VXU5	IFGGC3	Interferon-gamma-inducible GTPase IFGGC3 protein	468.000	266.580	0.816	0.009	Up
A0A286XG55	CPN2	Carboxypeptidase N subunit 2	762.330	538.770	0.496	0.011	Up
A0A286XCF3	LOC100724332	Peptidase S1 domain-containing protein	380.270	162.930	1.220	0.004	Up
A0A286Y257	PSME2	Proteasome activator subunit 2	853.100	597.430	0.516	0.022	Up
H0W8T6	SERPINA6	Serpin family A member 6	2015.830	1224.880	0.722	0.015	Up
A0A286XNN0	PTPN6	Tyrosine-protein phosphatase non-receptor type, EC 3.1.3.48	925.020	557.700	0.731	0.008	Up
H0VVP2	CLU	Clusterin	225.600	155.350	0.536	0.003	Up
A0A286XMG3	LOC100714630	GB1/RHD3-type G domain-containing protein	81.650	57.950	0.496	0.026	Up
A0A286XL53	FBLN1	Fibulin-1	660.330	483.950	0.444	0.032	Up
H0VE54	NAMPT	Nicotinamide phosphoribosyltransferase, NAmPRTase, EC 2.4.2.12	1115.530	720.300	0.632	0.016	Up
A0A286XFJ4		Uncharacterized protein	820.100	561.500	0.546	0.042	Up
H0VKX9	C8B	Complement component C8 beta chain (Complement component 8 subunit beta)	306.330	210.350	0.546	0.011	Up
H0UT		SERPIN domain-containing protein	277.680	207.550	0.422	0.005	Up

W7							
Q04962	F12	Coagulation factor XII, EC 3.4.21.38 (Hageman factor, HAF) [Cleaved into: Coagulation factor XIIa heavy chain; Coagulation factor XIIa light chain]	1739.280	900.970	0.949	0.037	Up
H0VKM0	NUB1	Negative regulator of ubiquitin like proteins 1	835.270	518.450	0.687	0.010	Up
H0UTL6	ARHGDIB	Rho GDP dissociation inhibitor beta	1367.800	738.450	0.888	0.013	Up
A0A286XYF7	C6	Complement component C6	106.050	77.580	0.454	0.006	Up
H0W145	ISG15	ISG15 ubiquitin like modifier	396.750	154.120	1.362	0.022	Up
H0VEH6	LOC100714630	GB1/RHD3-type G domain-containing protein	822.800	429.730	0.934	0.005	Up
H0WE16	UPP1	Uridine phosphorylase, EC 2.4.2.3	809.220	420.320	0.949	0.009	Up
H0UU63	GMFG	Glia maturation factor	81.580	48.020	0.766	0.021	Up
H0W5Q4	PGLYRP2	Peptidoglycan recognition protein 2	720.450	415.600	0.791	0.003	Up
A0A286XWT7	ME2	Malic enzyme	105.020	81.150	0.367	0.049	Up
H0V9N6	CFP	Properdin	560.380	358.000	0.651	0.024	Up
H0V129	PLEK	Pleckstrin	637.470	234.320	1.444	0.003	Up
H0UXU7	LOC100714902	GB1/RHD3-type G domain-containing protein	252.770	109.850	1.202	0.003	Up
A0A286XUG1	RANBP1	RAN binding protein 1	266.880	204.400	0.390	0.042	Up
H0W2B0		Serpin family G member 1	387.170	256.750	0.595	0.011	Up
H0VKW6	EIF2AK2	Eukaryotic translation initiation factor 2 alpha kinase 2 (Interferon-induced double-stranded RNA-activated protein kinase)	456.820	307.770	0.566	0.017	Up
H0VZY3	F9	Coagulation factor IX, EC 3.4.21.22 (Christmas factor)	161.900	104.070	0.642	0.017	Up
H0VMC7	IST1	IST1 homolog (Charged multivesicular body protein 8)	437.500	345.700	0.345	0.048	Up

A0A286XYS6	BTF3L4	Transcription factor BTF3	185.180	127.220	0.546	0.036	Up
A0A286XWP2	NMI	N-myc and STAT interactor	111.400	52.980	1.070	0.009	Up
A0A286XFI2	HSPBP1	HSPA (Hsp70) binding protein 1	36.200	30.080	0.263	0.037	Up
A0A286XQW9	FNBP1	Formin binding protein 1	385.250	258.700	0.575	0.007	Up
H0V7P7	HCLS1	Hematopoietic cell-specific Lyn substrate 1	219.880	167.750	0.390	0.045	Up
A0A286XW79	Tpd52	Tumor protein D52	87.420	64.620	0.433	0.030	Up
P49255	PTX2	Serum amyloid P-component, SAP	545.400	371.720	0.556	0.010	Up
A0A286XHI2		COesterase domain-containing protein	647.180	350.200	0.888	0.042	Up
H0VPX9		Haptoglobin	493.900	309.320	0.678	0.036	Up
A0A286XH52	CNN2	Calponin	209.370	145.400	0.526	0.048	Up
A0A286XXT2	C1QA	Complement C1q subcomponent subunit A	214.850	130.400	0.722	0.018	Up
H0UVJ2	NPC2	NPC intracellular cholesterol transporter 2 (Epididymal secretory protein E1)	269.700	197.230	0.454	0.011	Up
H0W265	CTSZ	Cathepsin X, EC 3.4.18.1	227.100	167.000	0.444	0.034	Up
H0V0A8	PLS1	Plastin 1	262.300	186.730	0.485	0.011	Up
H0UTI6	SNX1	Sorting nexin-1	96.530	66.200	0.546	0.018	Up
A0A286XZL9	AZGP1	Alpha-2-glycoprotein 1, zinc-binding	444.880	310.880	0.516	0.015	Up
A0A286XN08	NFIA	Nuclear factor 1	148.600	201.450	-0.434	0.026	Down
A0A286XSX9	MARCKSL1	MARCKS like 1	258.450	162.200	0.669	0.019	Up
H0V456	PSTPIP2	Proline-serine-threonine phosphatase interacting protein 2	75.470	51.750	0.546	0.023	Up
H0UXK2	CTSH	Cathepsin H	256.050	184.230	0.475	0.038	Up
A0A286XTY6	ARRB1	Arrestin beta 1	160.600	122.430	0.390	0.013	Up
H0W33	LGA	Galectin 3 binding protein	296.980	180.550	0.714	0.011	Up

3	LS3BP						
H0UZJ6	PDLIM1	PDZ and LIM domain 1	214.700	166.400	0.367	0.028	Up
H0VBL8	C1QB	Adiponectin A (Complement C1q B chain)	250.170	155.550	0.687	0.012	Up
H0VTV3		Uncharacterized protein	260.200	117.730	1.144	0.016	Up
A0A286XD20	TAX1BP3	Tax1-binding protein 3	157.620	119.170	0.401	0.023	Up
A0A286XGH1	SGCA	Sarcoglycan alpha	57.300	46.380	0.310	0.048	Up
J7PCK5	IFGGD2	Interferon-gamma-inducible GTPase IFGGD2 protein	121.650	94.100	0.367	0.014	Up
H0UXT9	RBP4	Apolipoprotein D, Apo-D	308.020	183.500	0.748	0.004	Up
H0V7X6	STK10	Non-specific serine/threonine protein kinase, EC 2.7.11.1	189.000	135.300	0.485	0.046	Up
H0VHZ4	ATP6V0D1	V-type proton ATPase subunit	335.800	206.600	0.705	0.022	Up
H0V077		Vitronectin	445.200	175.730	1.339	0.014	Up
A0A286X8S4	OAS1	2'-5' oligoadenylate synthase, EC 2.7.7.84	165.550	96.470	0.782	0.013	Up
H0VA43	ERO1A	Endoplasmic reticulum oxidoreductase 1 alpha	106.730	82.270	0.379	0.002	Up
H0VPR6	FCN1	Ficolin 1	267.250	147.280	0.856	0.004	Up
H0V4Y6	CHI3L1	Chitinase-3-like protein 1	118.850	79.580	0.575	0.021	Up
H0VV85	CASP10	Caspase 10	80.250	54.800	0.546	0.027	Up
H0VFB0	S100A11	Protein S100 (S100 calcium-binding protein)	1136.030	764.700	0.575	0.032	Up
H0VNM7	LNPEP	Leucyl and cystinyl aminopeptidase	60.050	49.600	0.275	0.020	Up
A0A286Y115	GIT2	Arf-GAP domain-containing protein	190.380	149.030	0.356	0.046	Up
A0A286XCT1	CXCL10	C-X-C motif chemokine ligand 10	233.250	106.050	1.138	0.010	Up
H0VI18	TNFAIP2	TNF alpha induced protein 2	50.420	29.520	0.774	0.017	Up
H0VT12	FLOT2	Flotillin	67.120	50.700	0.401	0.023	Up

A0A286Y400		F-box protein 6	167.480	97.600	0.782	0.023	Up
A0A286XES5	IGFALS	Insulin like growth factor binding protein acid labile subunit	50.480	29.620	0.766	0.004	Up
A0A286XK05		Serum amyloid A protein	106.770	70.170	0.604	0.003	Up
H0VZC4	LRG1	Leucine rich alpha-2-glycoprotein 1	206.380	138.150	0.575	0.040	Up
H0VLI4	AFP	Alpha fetoprotein	653.550	455.000	0.526	0.042	Up
H0VG12	MTHFD1L	Formate--tetrahydrofolate ligase, EC 6.3.4.3	117.400	94.730	0.310	0.027	Up
H0WE24	PARP14	Poly [ADP-ribose] polymerase, PARP, EC 2.4.2.-	164.150	106.930	0.623	0.017	Up
A0A286XDX4		Ig-like domain-containing protein	217.850	182.300	0.263	0.018	Up
H0UUA3	HMGB3	Uncharacterized protein	38.230	25.950	0.556	0.026	Up
H0V049		Uncharacterized protein	457.450	238.720	0.941	0.004	Up
A0A286XS98		LIM zinc-binding domain-containing protein	122.550	70.950	0.791	0.044	Up
H0VHG7	CTCF	CCCTC-binding factor	45.480	55.380	-0.286	0.008	Down
A0A286Y4S2	LOC100722668	Uncharacterized protein	122.780	74.670	0.714	0.007	Up

Supplementary Table S3. DEPs in PPD induration vs. EC erythema.

Protein IDs	Gene names	Protein names	PPD induration average	EC erythema average	log_2(FC)	p-value	Regulated Type
A0A286XV95		IF rod domain-containing protein	43208.820	25035.250	0.791	0.046	Up
A0A286Y5V6		IF rod domain-containing protein	3233.280	1530.520	1.077	0.015	Up
A0A286XLL1	SPTAN1	Spectrin alpha, non-erythrocytic 1	74.200	56.080	0.401	0.027	Up
A9QUC5	FABP5	E-FABP (Fatty acid binding protein 5)	2825.200	1934.720	0.546	0.041	Up
A0A286XX81	CSRP1	Cysteine and glycine rich protein 1	1348.570	1094.350	0.299	0.039	Up
H0VA29	ALAD	Delta-aminolevulinic acid dehydratase, EC 4.2.1.24	560.050	424.050	0.401	0.031	Up

A0A286XLN5	TAGLN	Transgelin	1979.220	1265.830	0.642	0.038	Up
A0A286XQ08		60S ribosomal protein L27a	591.250	441.420	0.422	0.041	Up
A0A286X9U8	RPL14	60S ribosomal protein L14	393.880	317.120	0.310	0.028	Up
A9LPA2	Cryab	Alpha-crystallin B chain (Alpha(B)-crystallin)	328.150	435.730	-0.415	0.046	Down
H0V391	SNRNP40	Small nuclear ribonucleoprotein U5 subunit 40	230.050	191.430	0.263	0.027	Up
H0V9C6	HMGCS1	Hydroxymethylglutaryl-CoA synthase, HMG-CoA synthase, EC 2.3.3.10 (3-hydroxy-3-methylglutaryl coenzyme A synthase)	345.700	259.480	0.411	0.038	Up
H0VR62	PEPD	Peptidase D	68.750	48.780	0.496	0.048	Up
H0UUM2	LOC100731677	Lysozyme, EC 3.2.1.17 (1,4-beta-N-acetylmuramidase C)	190.300	104.800	0.864	0.020	Up
